# Compression Fractures and Partial Phenotype Rescue With a Low Phosphorus Diet in the *Chihuahua* Zebrafish Osteogenesis Imperfecta Model

**DOI:** 10.3389/fendo.2022.851879

**Published:** 2022-02-24

**Authors:** Silvia Cotti, Ann Huysseune, Daria Larionova, Wolfgang Koppe, Antonella Forlino, Paul Eckhard Witten

**Affiliations:** ^1^Evolutionary Developmental Biology Group, Department of Biology, Ghent University, Gent, Belgium; ^2^Biochemistry Unit, Department of Molecular Medicine, University of Pavia, Pavia, Italy; ^3^SimplyFish AS, Stavanger, Norway

**Keywords:** osteogenesis imperfecta (OI), zebrafish, vertebral column deformities, compression fractures, osteoblasts, osteoclasts, collagen, dietary phosphorus

## Abstract

Osteogenesis imperfecta (OI) is a group of heritable disorders affecting bone and other connective tissues. Dominant OI forms are mainly caused by mutations in collagen type I. Patients suffer from skeletal deformities, fractures of long bones and vertebral compression fractures from early childhood onward. Altered collagen structure and excess mineralisation are the main causes for the bone phenotype. The *Chihuahua* (*Chi*/+) zebrafish has become an important model for OI. Given that reduced dietary phosphorus (P) intake reduces the bone mineral content and promotes bone matrix formation in teleosts, including zebrafish, we tested whether a low dietary P (LP) intake mitigates the OI phenotype in the *Chi/+* model. To answer this question, we characterised the *Chi/+* vertebral column phenotype at a morphological, cellular and subcellular level. We present the first description of vertebral compression fractures in *Chi/+* and assess the effects of LP diet on the *Chi/+* phenotype (*Chi*/+_LP_). Compared to untreated *Chi/+*, two months of LP dietary treatment decreases vertebral deformities in the abdominal region and reduces shape variation of caudal vertebral bodies to a condition more similar to wild type (WT). At the histological level, the osteoid layer, covering the bone at the vertebral body endplates in WT zebrafish, is absent in *Chi/+*, but it is partially restored with the LP diet. Whole mount-stained specimens and histological sections show various stages of vertebral compression fractures in *Chi/+* and *Chi*/+_LP_ animals. Both *Chi/+* and *Chi/+*_LP_ show abundant osteoclast activity compared to WT. Finally, the ultrastructure analysis of WT, *Chi/+* and *Chi*/+_LP_ shows *Chi/+* and *Chi*/+_LP_ osteoblasts with enlarged endoplasmic reticulum cisternae and a high protein content, consistent with intracellular retention of mutated collagen. Nevertheless, the secreted collagen in *Chi*/+_LP_ appears better organised concerning fibre periodicity compared to *Chi/+*. Our findings suggest that a reduced mineral content of *Chi/+* bone could explain the lower frequency of vertebral column deformities and the restored shape of the vertebral bodies in *Chi*/+_LP_ animals. This, together with the improved quality of the bone extracellular matrix, suggests that two months of reduced dietary P intake can alleviate the severe bone phenotype in *Chi/+* zebrafish.

## Introduction

Osteogenesis imperfecta (OI), also known as ‘brittle bone disease’, is a clinically and genetically heterogeneous group of heritable disorders affecting bone and other connective tissues with collagen type I as main matrix component (1). Clinical features of OI patients are short stature, skeletal deformities, low bone mass and bone fragility. Frequent fractures also in absence of trauma can occur *in utero* and cause death before birth (2, 3). Scoliosis and compression fractures of the vertebral bodies are the most severe complications in human patients (4, 5). Vertebral compression fractures require surgery to stabilise the vertebral column and treatment with bisphosphonates to prevent bone loss (6–8).

In the majority of patients, OI is caused by autosomal dominant mutations in *COL1A1* and *COL1A2*, coding for α1 and α2 chains of the collagen type I (9). These OI forms, regarded as classical OI, were first classified in 1979 by Sillence and co-workers in 4 different types of OI (OI I-IV) based on clinical observations, radiographic features and the mode of inheritance (2). The types range from mild (type I), over moderate (type IV), severe (type III) to perinatally lethal (type II). The mildest form, OI type I, is caused by quantitative deficiency of structurally unaltered collagen. Patients have a normal or slightly short stature, deformities of long bones in the legs and are susceptible to bone fractures from early on. The moderate, severe and lethal OI forms are caused instead by alterations in the collagen structure. The most common mutations causing structural alterations of collagen type I are single-nucleotide variants that substitute glycine with a bulkier or with a charged residue within the Gly-X-Y repeat, either in the α1 or α2 chains (9). This results in delayed collagen folding and excess post-translational modifications (10).

Zebrafish (*Danio rerio*) has become an important model organism for the study of human skeletal disorders, due to its high reproduction rate and easy access to embryos. Moreover, basic processes of skeletal formation are conserved across gnathostomes (11–14). The first identified zebrafish mutant model for classical dominant OI is the *Chihuahua* (*Chi/+*). The mutant was isolated from a large N-ethyl-N-nitrosourea (ENU)-mutagenesis screen for skeletal dysplasias (15). *Chi/+* carries a heterozygous glycine to aspartate substitution in position 736 in the α1 chain of collagen type I. Adult animals display typical OI characters, such as bending of the vertebral column, bone fragility (rib fractures), high bone mineral-to-matrix ratio and reduced bone elasticity (16, 17).

Next to structural alterations in collagen type I, also hypermineralisation leads to fragility and poor bone quality, both in OI patients and in zebrafish models (17). It is a common assumption that an increase in bone mineral content enhances the mechanical properties of bone. Proper mechanical function of bone requires, however, a balance between toughness, provided by the collagenous matrix, and stiffness, provided by the mineral phase (18). The antlers of deers and elks are examples for bones that withstand extreme mechanical forces. Yet, the bone mineral content of antlers is about half of that of a human femur (19). Excess mineralisation, on the other hand, makes bone stiff and brittle (18) and increases the risk of fractures (20, 21). Thus, while mutations in OI patients cannot be undone, lowering the bone mineral content could possibly alleviate skeletal defects in these patients. Recent studies on teleost fish, including wild type (WT) zebrafish (22), as well as farmed Atlantic salmon (23–25), have shown that periods of severely lowered dietary phosphorus (P) intake (-50%) reduce the bone mineral content and promote the formation of non-mineralised bone without causing vertebral column malformations. In contrast, but similar to OI patients and animal models, zebrafish with increased dietary P intake and animals with age-related increased bone mineral density show elevated rates of vertebral column malformations (22, 26).

We have used these new insights to investigate if a reduction of dietary P intake can mitigate deformities of the vertebral column in adult *Chi/+* zebrafish. To answer this question we have characterised the *Chi/+* vertebral column phenotype at a morphological, cellular and subcellular level and assessed the effect of a low P (LP) diet on the *Chi/+* phenotype (further referred to as *Chi*/+_LP_). The LP diet decreases vertebral deformities in the abdominal region and partially restores shape variation of the caudal vertebral bodies in the *Chi/+* mutant. The osteoid layer covering the bone at the endplates in WT is absent in the *Chi/+* mutants, but partially restored with the LP diet. Vertebral body fractures are observed in animals of both groups, *Chi/+* and *Chi*/+_LP_. In both groups, the bone shows evidence of fracture repair and remodelling, supported by the observation of abundant osteoclast activity in *Chi/+* compared to WT fish. Finally, both *Chi/+* and *Chi*/+_LP_ display osteoblasts with enlarged endoplasmic reticulum (ER) cisternae and a high protein content consistent with intracellular retention of defective collagen. The secreted collagen in *Chi*/+_LP_ appears, nevertheless, better organised concerning fibre periodicity than *Chi/+*.

## Results

### *Chihuahua* Mutants Have Increased Frequency of Vertebral Column Deformities

**Figure 1:**
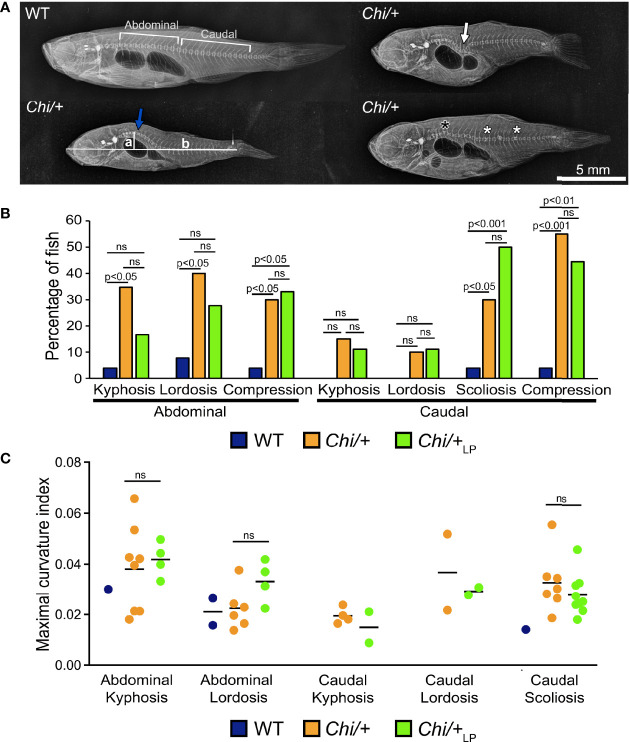
Vertebral column deformities in *Chi/+* and *Chi*/+_LP_ mutants. **(A)** Representative X-rays of three months old WT and *Chi/+* (representative also for the malformations diagnosed in *Chi*/+_LP_) zebrafish show severe vertebral column deformities in mutants, i.e. abdominal kyphosis (blue arrow), abdominal lordosis (white arrow), vertebral body compressions (white asterisks) and hemivertebra (black asterisk). **(B)** Frequency of malformations. *Chi/+* mutants (n=20) show increased frequency of kyphosis, lordosis, scoliosis and vertebral body compressions in the abdominal and caudal vertebral column compared to WT animals (n=26). *Chi*/+_LP_ animals (n=18) display reduced kyphosis and lordosis of the abdominal, but increased scoliosis of the caudal vertebral column. Alterations were diagnosed based on Alizarin red S whole mount-stained specimens; only the abdominal and caudal region of the vertebral column were considered for the analysis. Chi-squared test followed by Bonferroni correction; p values are indicated; ns: non-significant. **(C)** Severity assessment of the maximal curvature index diagnosed in WT, *Chi/+* and *Chi*/+_LP_ related to abdominal kyphosis and lordosis, and to caudal kyphosis, lordosis and scoliosis. The graph shows individual data points and the mean value (black bar) for the maximal curvature index. The maximal kyphotic and lordotic indices were calculated in the sagittal plane of Alizarin red S stained specimens as the ratio (a/b) between the perpendicular distance from the axis (in correspondence of the maximal curvature, segment ‘a’ in **A**) and the standard length (segment ‘b’ in **A**). The same method but in the coronal plane was used to calculate the maximal scoliotic index. Mann-Whitney test was applied with a minimum of three values per group; ns, non-significant.

Starting from 28 days post-fertilisation (dpf), WT were fed a regular P diet for two months. At 28 dpf, *Chi/+* mutants were randomly divided in two groups and fed a ‘LP diet’ (low P content, *Chi*/+_LP_) or a regular P diet (*Chi/+*) for two months. Three months old *Chi/+* show severe deformities compared to WT fish on Faxitron X-ray images (**Figure 1A**), confirming earlier reports (15–17). Vertebral column deformities of WT, *Chi/+* and *Chi*/+_LP_ were assessed by whole mount Alizarin red S staining (details are described below). Compared to WT, *Chi/+* mutants have an increased frequency of kyphosis and lordosis in the abdominal region of the vertebral column. Kyphosis and lordosis are reduced in the abdominal region of the vertebral column in *Chi*/+_LP_ fish. However, compared to *Chi/+* fish, *Chi*/+_LP_ fish show increased scoliosis in the caudal vertebral column. Both *Chi/+* and *Chi*/+_LP_ have a high frequency of compressed vertebral bodies; examples are shown in **Figure 1A**, frequency of deformities is shown in **Figure 1B**. To assess the severity of the curvature in those specimens diagnosed with kyphosis, lordosis and scoliosis, the maximal kyphotic, lordotic and scoliotic indices were measured, respectively. **Figure 1C** shows the maximal curvature indices in the abdominal and caudal region of affected WT, *Chi/+* and *Chi*/+_LP_ individuals. No statistical differences in the degree of curvature are detected between *Chi/+* and *Chi*/+_LP_ fish at three months of age (Mann-Whitney test: non-significant).

The general metrics for the analysis of the vertebral column malformations (**Table 1**) show that the frequency of specimens with at least one malformation is 19% in WT, 75% in *Chi/+* and 89% in *Chi*/+_LP_. The highest average malformation load is in the *Chi*/+ group (3.27 malformations/deformed specimen) where a total of 49 malformations were identified. The average malformation load in the *Chi*/+_LP_ group is 2.5 with a total of 40 diagnosed malformations. These data indicate a tendency towards reduced number of malformations and average malformation load in the *Chi*/+_LP_ compared to untreated *Chi*/+ (Chi-square test: p=0.27).

**Table 1:** General metrics for the analysis of vertebral column malformations in WT, *Chi/+* and *Chi/+*_LP_.

	WT	*Chi/+*	*Chi/+*_LP_
Number of observed specimens	26	20	18
Total number of malformations	6	49	40
Frequency (%) of specimens with at least one malformation	19	75	89
Average malformation load	1.20	3.27	2.5

### Partial Rescue of Vertebral Body Shape Variation in *Chihuahua* Mutants Under the LP Diet

**Figure 2:**
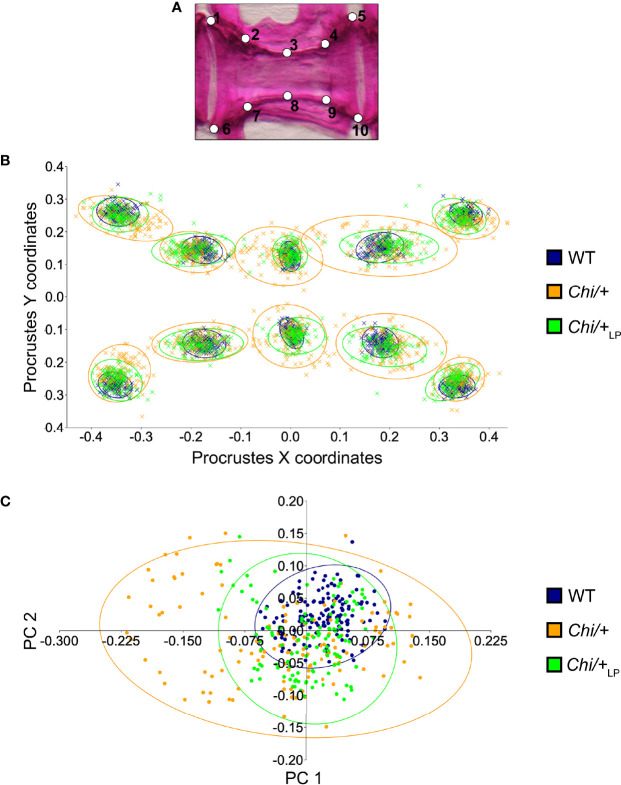
Vertebral body shape variation in *Chi/+* and *Chi*/+_LP_ mutants. **(A)** Alizarin red S stained vertebral body of a WT animal with 2D landmark positions used for quantifying the shape variation by means of geometric morphometrics, represented in **(B)**. **(B)** The scatterplot of WT, *Chi/+* and *Chi*/+_LP_ 2D landmarks shows high variation in the superimposition of X,Y Procrustes coordinates of *Chi/+* compared to WT animals. *Chi*/+_LP_ animals display reduced landmark variation compared to *Chi/+* and a distribution more similar to WT indicating a partial rescue of shape variation at three months of age. The first 10 caudal vertebral centra in WT (n=15), *Chi/+* (n=13) and *Chi*/+LP (n=12) were analysed. The 95% confidence ellipses are shown. **(C)** Principal component analysis of vertebral centra shapes. Each symbol in the plot represents a vertebral body. PC indicates Principal Component and the values in the axis labels indicate the percentage of variation accounted for by each axis. *Chi/+* animals show high variance compared to WT animals (*Chi/+* versus WT, PC1 0.3321, PC2 0.1795; Chi-square test: p < 0.001). Variance is rescued in *Chi*/+_LP_ animals (*Chi*/+_LP_ versus WT, PC1 0.2484, PC2 0.2020; Chi-square test: non-significant). The 95% confidence ellipses are shown.

To quantify shape variation of the caudal vertebral centra in WT, *Chi/+* and *Chi*/+_LP_ animals, landmark-based geometric morphometrics was applied, based on whole mount-stained specimens (**Figure 2A**). The scatterplot of 2D landmarks shows differences in location of corresponding landmarks between WT, *Chi/+* and *Chi*/+_LP_ animals. The scatterplot shows a high variation in the superimposition of Procrustes coordinates of *Chi/+* compared to WT. *Chi*/+_LP_ display reduced variation of landmarks compared to *Chi/+* and a distribution more similar to WT (**Figure 2B**). Principal component analysis (PCA) of superimposed landmarks shows the amount of variation between WT, *Chi/+* and *Chi*/+_LP_. Only principal component 1 (PC1) and principal component 2 (PC2) were considered. Compared to WT, *Chi/+* show a statistically significant higher variance (*Chi/+* versus WT, PC1 0.3321, PC2 0.1795; Chi-square test: p<0.001). On the contrary, *Chi*/+_LP_ animals show a non-statistically different variance compared to WT (*Chi*/+_LP_ versus WT, PC1 0.2484, PC2 0.2020; Chi-square test: non-significant) (**Figure 2C**).

**Figure 3:**
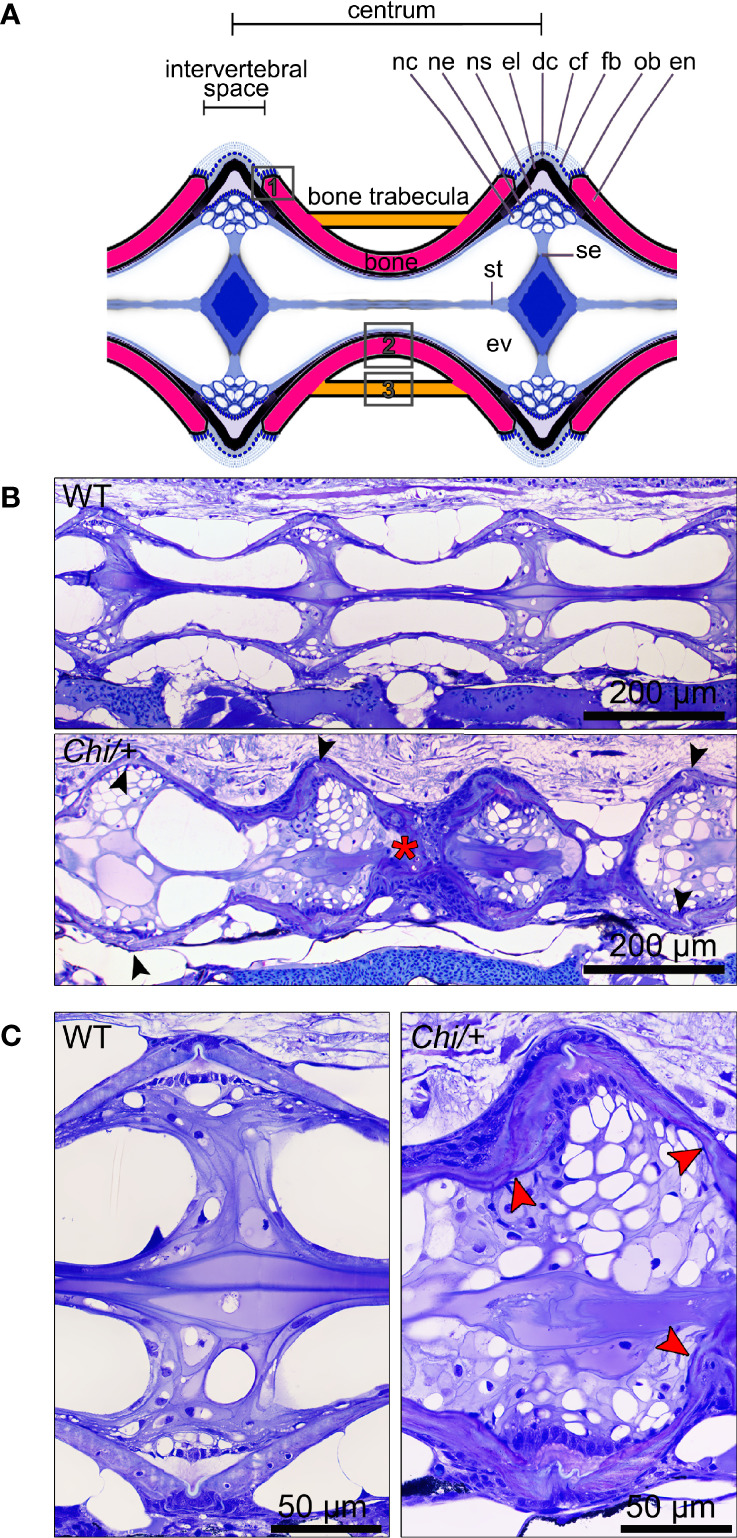
Histology of vertebral column of WT, *Chi/+* and *Chi*/+_LP_ confirms the irregular shape of mutant vertebral bodies. **(A)** Schematic representation of the medio-sagittal plane of a zebrafish vertebral body centrum and two intervertebral spaces. Vertebral centra derive from segmental mineralisation of the notochord sheath and intramembranous bone formation around the notochord. Vertebral body endplates (en) are connected by intervertebral ligaments. Ligaments consist of the enlarged notochord sheath (ns, a collagen type II layer secreted by the cells of the notochord epithelium, ne), its outer elastin layer (el) and dense collagen type I fibre bundles (dc) produced by fibroblasts (fb) that surround the notochord. The collagen type I fibre bundles (cf) continue in the bone of the vertebral body endplates (en) as Sharpey fibres. Osteoblasts (ob) deposit new bone matrix that expands the vertebral body endplates in the bone growth zone. Inside, the notochord is composed of vacuolated notochord cells (nc) and extracellular vacuoles (ev). Condensed notochord cells constitute the notochord septum (se) and the notochord strand (st). Boxes indicate locations where the bone thickness was measured, i.e. endplates (1), central region of vertebrae (2) and trabecular bone (3). **(B)** Representative three months old WT and mutant sagittal sections of the vertebral column stained with toluidine blue. Compared to WT, Chi/+ mutants (representative also for *Chi*/+_LP_) have several vertebral centra with deformed endplates that are shifted against each other along the dorsal-ventral axis (black arrowheads). *Chi/+* animals also suffer from vertebral body compression fractures (red asterisk), scoliosis, lordosis and kyphosis. Scoliosis can be appreciated from the absence of a straight sagittal midline plane as seen in the WT animal. **(C)** Higher magnification of vertebral body endplates in WT and *Chi/+* (representative also for *Chi*/+_LP_) animals. Toluidine blue staining shows deformed endplates of adjacent vertebral bodies (red arrowheads) in *Chi/+* mutants, yet with unaltered ligaments and unaltered intervertebral space as in WT.

Histological assessment of the vertebral column in the sagittal plane confirmed the irregular shape of mutant vertebral bodies compared to WT vertebral bodies (**Figure 3**). Different from WT (**Figures 3A, B**), in *Chi/+* animals vertebral body endplates of adjacent vertebral bodies are frequently shifted against each other along the dorsal-ventral axis (**Figure 3B**). Moreover, scoliosis of the vertebral column in *Chi/+* and *Chi*/+_LP_ animals, previously observed on whole mount-stained specimens is visible on histological sections as the absence of a sagittal middle plane (**Figure 3B**). Histology reveals the presence of compression fractures in *Chi/+* and *Chi*/+_LP_ animals (see below for details) (**Figure 3B**). Vertebral centra of *Chi/+* and *Chi*/+_LP_ animals display distortions of the endplates not observed in WT zebrafish (**Figure 3C**). Despite these distortions, the endplates retain structurally unaltered intervertebral spaces with unaltered ligaments as in WT animals (**Figure 3C**).

### *Chihuahua* Vertebrae Are Thin, Highly Mineralised and Lack an Osteoid. LP Diet Restores the Osteoid

**Figure 4:**
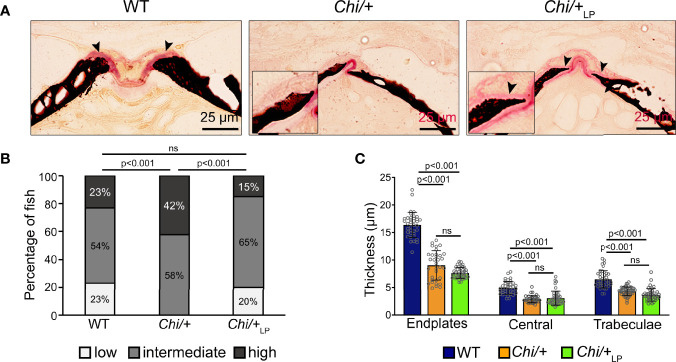
*Chi/+* vertebral bone structures are thin and highly mineralised. The LP diet restores the osteoid. **(A)** Sagittal histological non-demineralised sections stained with Von Kossa/Van Gieson show that three months old *Chi/+* animals, compared to WT animals, have highly mineralised endplates. No osteoid layer can be identified. The osteoid (pink, black arrowheads) is restored in *Chi*/+_LP_. Mineralised bone: black; dense collagen and non-mineralised bone: red. **(B)** Quantitative analysis of vertebral body endplate mineralisation (scored as low, intermediate or high) based on whole mount-stained specimens shows that *Chi/+* animals exhibit a higher degree of mineralisation compared to WT animals. The LP diet reduces mineralisation of the vertebral body endplates in some *Chi/+* individuals. The first 5 caudal vertebral centra in WT (n=15), *Chi/+* (n=13) and *Chi*/+_LP_ (n=12) were analysed. Chi-square test followed by Bonferroni correction, p values are indicated, ns: non-significant. **(C)** Measurements of bone structure thickness at three locations: (i) vertebral endplates, (ii) central region of vertebrae and (iii) trabecular bone (see **Figure 3A** for locations). Compared to WT animals, *Chi/+* and *Chi*/+_LP_ animals have thinner bone structures in all three locations (see also **Table 2**). Thickness of bone structures was measured on toluidine blue stained sections in 5 to 10 vertebral centra in WT (n=4), *Chi/+* (n=4) and *Chi*/+_LP_ (n=5). Mann-Whitney test followed by Bonferroni correction, p values are indicated, ns: non-significant.

Histological sections of non-demineralised vertebrae in the sagittal plane stained with Von Kossa/Van Gieson show the absence of a detectable osteoid layer (non-mineralised new bone matrix) in the growth zone of the vertebral endplates in *Chi/+* animals (**Figure 4A**). In *Chi*/+_LP_ animals the non-mineralised osteoid layer is restored (**Figure 4A**).

Vertebral body endplate mineralisation was assessed quantitatively based on the extent of the mineralised and non-mineralised bone matrix in whole mount specimens stained with Alizarin red S. In comparison to WT, *Chi/+* mutants show higher mineralisation of the vertebral body endplates (Chi-square test: p<0.001), but the LP diet is capable of partially reducing endplate mineralisation in some *Chi*/+_LP_ individuals (Chi-square test: *Chi*/+_LP_ versus Chi/+, p<0.001; *Chi*/+_LP_ versus WT, non-significant) (**Figure 4B**).

Finally, midline sections of the vertebral column were used to measure the thickness of bone structures in WT, *Chi/+* and *Chi*/+_LP_. Histomorphometry reveals that *Chi/+* and *Chi*/+_LP_ bone structures are significantly thinner compared to WT. This is the case for vertebral body endplates, the central region of the vertebral body and the trabecular bone (**Figure 4C**, **Table 2**).

**Table 2:** Bone histomorphometry.

		Thickness, Mean ± SD (μm)	Pairwise p-values	
Vertebral body endplates	WT	16.39 ± 2.27	WT - *Chi/+*	4.6 × 10^-12^
	*Chi/+*	9.03 ± 2.66	WT - *Chi*/+_LP_	7.1 × 10^-14^
	*Chi*/+_LP_	7.63 ± 0.98	*Chi*/+ - *Chi*/+_LP_	ns
Vertebral body central region	WT	4.95 ± 1.19	WT - *Chi/+*	3.5 × 10^-10^
	*Chi/+*	2.90 ± 0.68	WT - *Chi*/+_LP_	2.3 × 10^-8^
	*Chi*/+_LP_	3.10 ± 1.30	*Chi*/+ - *Chi*/+_LP_	ns
Trabecular bone	WT	6.50 ± 1.58	WT - *Chi/+*	3.5 × 10^-10^
	*Chi/+*	4.33 ± 0.78	WT - *Chi*/+_LP_	2.3 × 10^-8^
	*Chi*/+_LP_	3.66 ± 1.14	*Chi*/+ - *Chi*/+_LP_	ns

Statistical analysis is based on Mann-Whitney test followed by Bonferroni correction; ns, non-significant.

### Compression Fractures and Fracture Repair in *Chihuahua* Vertebral Bodies

**Figure 5:**
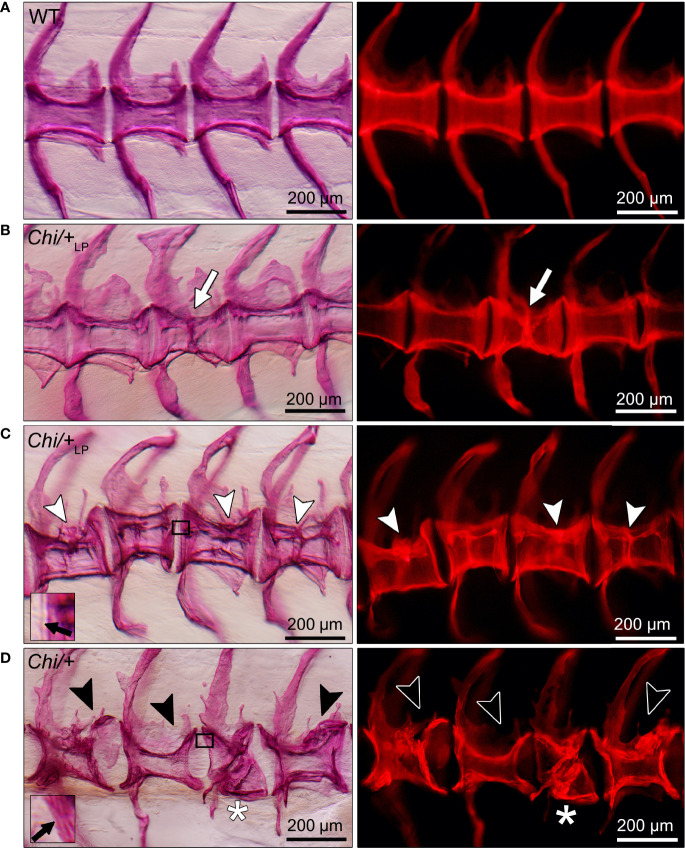
Different grades of *Chi/+* and *Chi*/+_LP_ compression fractures. Whole mount Alizarin red S stained vertebral bodies of WT **(A)** and mutants **(B–D)** visualised in bright field (left) and with fluorescence (right). Inter-individual variability and different severity levels of compression fractures are observed in three months old mutant zebrafish. Bone calli associated to fractures appear more dense than other bone elements when visualised with fluorescent light. **(B)** Example of a mutant *Chi*/+_LP_ showing a compression fracture affecting only one vertebral body, bone callus is visible (white arrow). **(C)** *Chi*/+_LP_ zebrafish displaying kyphosis associated with multiple compression fractures and evident bone calli (white arrowheads). **(D)** Mutant *Chi*/+ fish displaying severely distorted vertebrae (black arrowheads) and a collapsed vertebral body (white asterisk). The inserts in **(C, D)** demonstrate the identification of osteoid on whole mount-stained specimens. Black arrows indicate the presence **(C)** and absence **(D)** of osteoid in *Chi*/+_LP_ and *Chi*/+, respectively.

The analysis of whole mount-stained specimens reveals that *Chi/+* and *Chi*/+_LP_ animals have vertebral compression fractures that relate to the anteroposterior compression of vertebral centra (**Figure 5**). Compression fractures are absent in WT animals (**Figure 5A**). Different severity levels of compression fractures are observed in both *Chi/+* and *Chi*/+_LP_ individuals (**Figures 5B–D**). Some *Chi*/+_LP_ animals present compression fractures that only affect one vertebral body (**Figure 5B**). Some *Chi*/+_LP_ mutants show kyphosis associated with multiple compression fractures (**Figure 5C**). *Chi*/+ mutant fish display severely distorted vertebrae, as described above, and collapsed vertebral centra (**Figure 5D**). Regardless of the inter-individual variability among the fractures, the bone of fracture repair calli appears more dense than other bone elements when visualised with fluorescent light.

**Figure 6:**
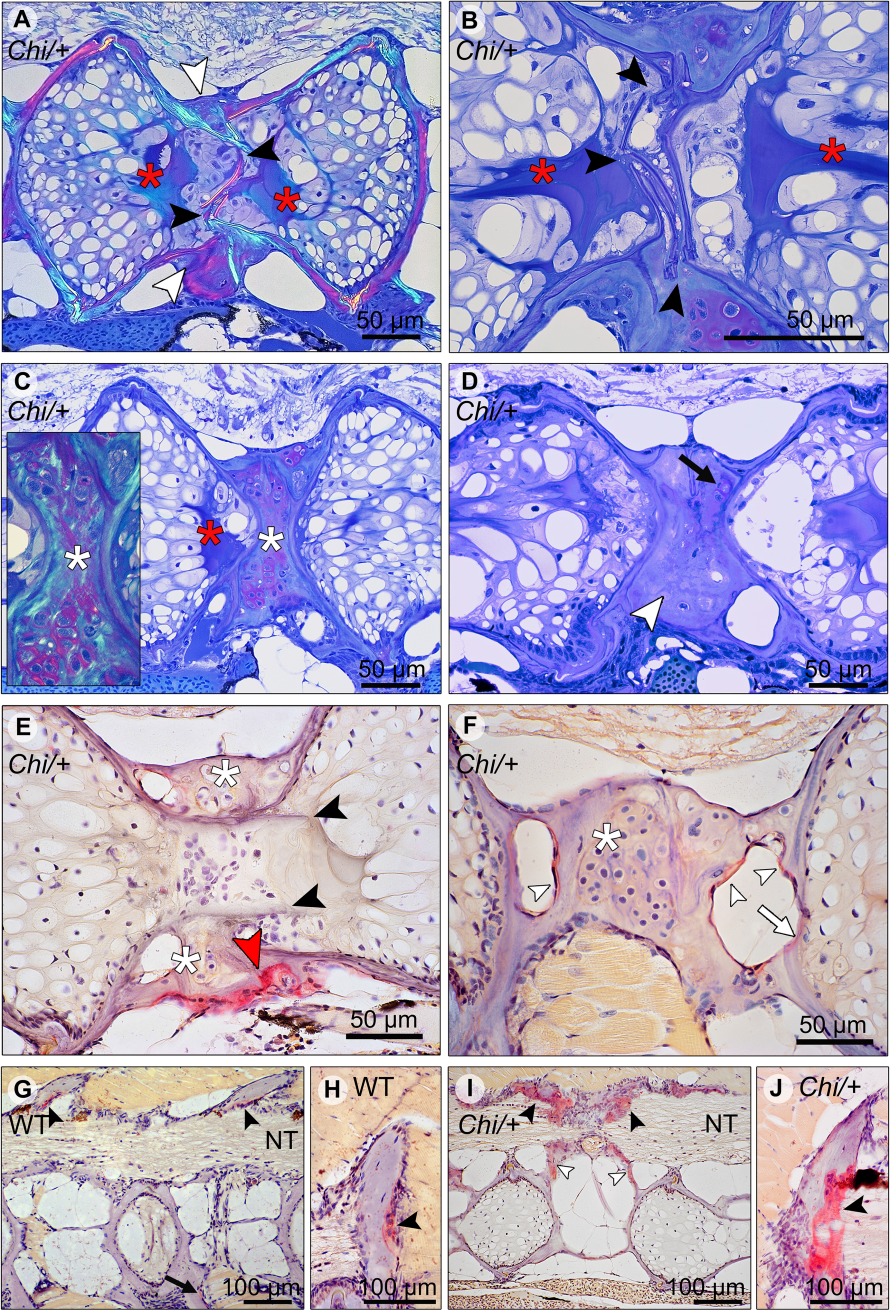
Compression fractures and bone resorption in *Chi/+* and *Chi*/+_LP_ vertebral bodies. **(A)** Toluidine blue stained medio-sagittal section of a compression fracture from three months old mutant zebrafish (representative for both *Chi*/+ and *Chi*/+LP) observed with polarised light. The compression fracture is characterised by several fractures in the central region of the vertebral body (black arrowheads); a bone callus is present on the outside of the vertebral centrum (white arrowheads). The fracture also disrupts the notochord tissue and induces condensation of chordocytes into a fibrous tissue (a known reaction of notochord tissue to injuries) (red asterisks). **(B)** High magnification of the fractured bone (black arrowheads) inside the notochord. The reaction of the notochord tissue can be seen (red asterisks). **(C)** A fibrocartilaginous callus is present around the fractured central part of the vertebral body, the typical appearance for fracture callus at initial stages of repair (white asterisks). Polarised light (insert) shows collagen fibres (green) within the cartilaginous callus. Red asterisk indicates the notochord tissue condensation. **(D)** Sagittal section of a healed compression fracture. Fracture repair and remodelling processes replaced the fibrocartilaginous tissue by a bone callus (white arrowhead). Remnants of the fibrocartilaginous tissue are visible (black arrow). **(E)** Tartrate-resistant acid phosphatase (TRAP) staining confirms compression fracture repair. TRAP activity (red staining, red arrowhead) indicates resorption of the fibrous tissue (white asterisks) that is being replaced by a hard bone callus. The fractured bone fragments (black arrowheads) in the lumen of the notochord do not show resorption, which is consistent with the absence of blood vessels, lymphatic vessels and innervation inside the notochord. **(F)** TRAP activity is detected also in the trabecular bone (white arrowheads) and vertebral endplate (white arrow) of a vertebra showing a compression fracture in mutant zebrafish (representative for both *Chi*/+ and *Chi*/+_LP_). White asterisk indicates the fibrocartilaginous callus. **(G, H)** WT display TRAP activity (red) at sites of bone remodelling linked to bone growth, i.e. the endosteal surfaces of the neural (black arrowheads) and haemal arches (black arrow). NT, neural tube. **(I, J)** *Chi/+* animals (representative also for *Chi*/+_LP_) show TRAP activity at the same locations as in WT, however mutants exhibit expanded TRAP activity at all endosteal and periosteal bone surfaces, i.e. arches (black arrowheads) and bone trabeculae connecting the endplates (white arrowheads). NT, neural tube.

Histological sections in the sagittal plane of mutant vertebral columns confirm the presence of compression fractures in *Chi/+* and *Chi*/+_LP_ animals (**Figure 6**). A variety of fractured sites suggests that compression factures range from being in a status of repair (as evidenced by bone remodelling, see below) to fractures that do not, or not yet, display evidence of repair. Compression fractures affect the central region of the vertebral bodies. The fractured bone trabeculae become displaced into the chordocyte-filled lumen of the notochord (**Figures 6A, B**). When fractures injure the notochord, the notochord strand and the notochord septum (see **Figure 3A** for the anatomical terms) become condensed [keratinised in response to tissue damage according to (26)] (**Figures 6A, B**). Analysis of serial sections from several *Chi/+* and *Chi*/+_LP_ animals reveals that compression fractures display variable degrees of repair. Fractured sites show fibrocartilaginous calli at the outer surface of the compressed vertebral bodies (**Figure 6C**). Repair evidently continues with replacement of fibrocartilage tissue by a bone callus (**Figure 6D**). Repair at fracture locations is further confirmed by the demonstration of tartrate-resistant acid phosphatase (TRAP). TRAP is produced by osteoclasts and secreted onto the bone surface at the locations of bone resorption. TRAP activity is also linked to the resorption of fibrocartilage that is being replaced by a bone callus (**Figure 6E**) (27). Osteoclast activity is also observed at the bone trabeculae and vertebral endplates of vertebral compression fractures (**Figure 6F**). No signs of fracture repair or bone resorption are detected on bone elements inside the notochord (**Figure 6E**). This agrees with the fact that the notochord contains neither blood vessels, nor nerve fibres and no lymphatic vessels (28).

### *Chihuahua* Mutants Have Increased Bone Resorption

Osteoclasts and locations of bone resorption (other than locations of fracture repair) in growing juvenile individuals were identified by the demonstration of TRAP. In WT zebrafish TRAP activity is typically restricted to locations within the vertebral column that require resorption for allometric growth (27), i.e. the endosteal surfaces of enlarging neural and haemal arches (**Figures 6G, H**). *Chi/+* and *Chi*/+_LP_ also display TRAP staining at the endosteal surfaces of the neural and haemal arches, but the enzymatic activity is increased and extends also to other bone structures such as the bone trabeculae (**Figures 6I, J**).

**Figure 7:**
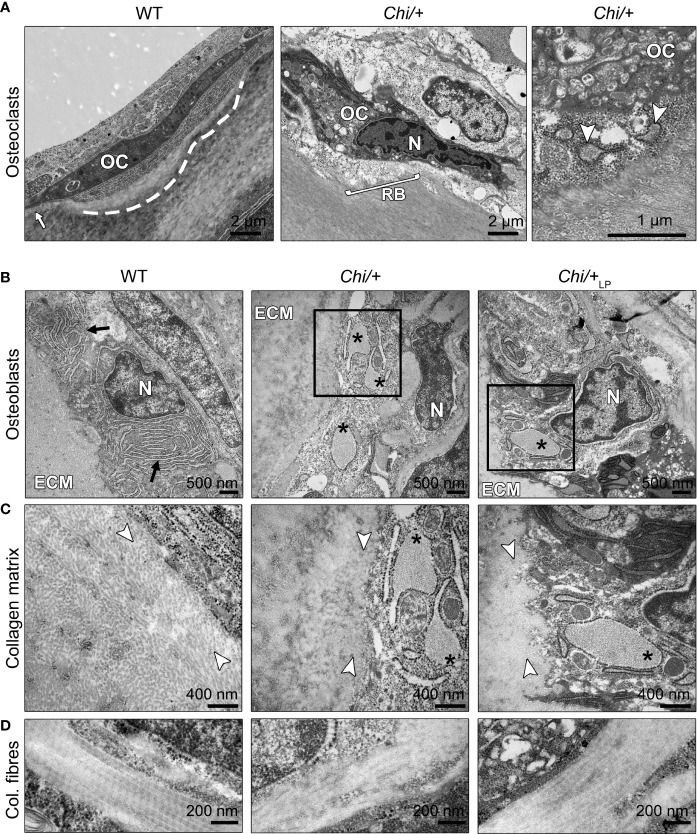
Ultrastructure of bone cells and bone matrix in *Chi/+* and *Chi*/+_LP_ animals. **(A)** Transmission electron microscopy (TEM) of osteoclasts (OC) located at the arch surface of three months old WT and *Chi/+* (representative for both *Chi*/+ and *Chi*/+_LP_) vertebrae. The panel WT shows a typical flat-shaped teleost osteoclast (OC) characterised by its electrodense cytoplasm. The cell resides in a shallow resorption lacuna (dashed line), yet is attached to the bone surface (white arrow). The panel *Chi/+* shows an osteoclast that resorbs the bone matrix and exhibits the typical ‘ruffled border’ (RB), an electron-dense cytoplasm with abundant cytoplasmic vacuoles and resorption vesicles (right panel, white arrowheads) in proximity of the ruffled border. N, nucleus. **(B)** TEM of WT, *Chi/+* and *Chi*/+_LP_ shows mutant osteoblasts with enlarged endoplasmic reticulum (ER) cisternae (asterisks). The osteoblasts are located in the growth zone of the vertebral body endplate (see **Figure 3A** for location). Higher magnification images in **(C)** show that *Chi/+* osteoblast ER cisternae are filled with protein, likely mutated collagen type I WT osteoblasts have numerous, yet not enlarged ER cisternae (arrows). ECM: extracellular matrix of the bone surface; N: nucleus. **(C)** The newly secreted collagen fibrils (white arrowheads) in the proximity of the osteoblasts in WT are visibly separated prior to maturation and assemblage into collagen fibres. In contrast, the *Chi/+* animal has densely packed collagen fibrils. No space can be recognised between the fibrils. In the *Chi*/+_LP_ individual collagen fibres are densely packed and space between the fibrils is distinguishable in proximity to the osteoblasts. **(D)** Longitudinal sections of collagen fibres show a regular D-periodicity pattern in a WT animal, absence D-period pattern in a *Chi/+* mutant, and a less regular D-periodicity pattern a *Chi*/+_LP_ specimen.

Transmission electron microscopy (TEM) confirms the presence of osteoclasts, attached to the endosteal surfaces of arches in WT, *Chi/+* and *Chi*/+_LP_ (**Figure 7A**). These cells, while attached to the bone surface, reside in resorption lacunae (as shown in **Figure 7A**, WT zebrafish). Actively resorbing osteoclasts exhibit a typical ‘ruffled border’, an electron-dense cytoplasm with abundant cytoplasmic vacuoles and resorption vesicles in proximity of the ruffled border (as shown in **Figure 7A**, *Chi/+* zebrafish).

### *Chihuahua* Mutants Show Signs of ER Stress and Altered Collagen Type I

The ultrastructure of bone cells and bone matrix in the vertebral column was analysed by TEM at the level of the medio-sagittal plane. Ultrathin sections of representative specimens show that *Chi/+* and *Chi*/+_LP_ have enlarged endoplasmic reticulum (ER) cisternae in osteoblasts located in the growth zone of the vertebral body endplates (**Figure 7B**, see **Figure 3A** for location). Likewise, ER cisternae in osteoblasts along the neural and haemal arches are enlarged. High magnification images show that *Chi/+* ER cisternae are filled with protein (**Figure 7C**). Conversely, WT osteoblasts have numerous, yet not enlarged ER cisternae, indicative for high protein synthesis activity at the growth zone of the endplates (**Figure 7B**).

The collagen matrix appears altered in the *Chi/+* mutants compared to WT. In WT, newly secreted collagen fibrils in the proximity of the osteoblasts (osteoid) are dispersed prior to maturation and assemble into larger fibres at a distance from the osteoblasts. In contrast, the *Chi/+* bone matrix is characterised by densely packed collagen fibrils in the vicinity of the osteoblasts (**Figure 7C**). In *Chi*/+_LP_, individual collagen fibrils are partly distinguishable in close proximity of the osteoblasts, indicative for less collagen packing and a more typical osteoid (**Figure 7C**).

Longitudinal sections of collagen fibres in WT zebrafish show a regular D-periodicity. The pattern is absent in *Chi/+* mutants. In *Chi*/+_LP_ the collagen D-periodicity is partially visible along the fibre (**Figure 7D**).

## Discussion

### *Chihuahua* Zebrafish Suffer From Vertebral Column Deformities, Low Dietary P Partially Rescues the Bone Phenotype

Early reports on children and young patients diagnosed with OI described severe vertebral column deformities as the major complications of the disease. From very young age onwards, patients suffer from vertebral body deformities associated with progressive scoliosis, kyphosis or kyphoscoliosis (2, 4, 29). This condition is known to be age-dependent and usually worsens after the age of six (4, 30). Likewise, *Chi/+* zebrafish display severe bending of the vertebral column, i.e. kyphosis and lordosis, at three months of age, as demonstrated by whole mount Alizarin red S staining and histological sections. Similar to human patients, such malformations progressively worsen in adult stages (16, 17). Vertebral body deformities, vertebral compressions and fractures represent other severe complications of young patients (4, 29). We show here for the first time that juvenile *Chi/+* zebrafish suffer from vertebral body compressions in both the abdominal and caudal region of the vertebral column. Mutant vertebral bodies are distorted and characterised by increased shape variation compared to WT, as revealed by geometric morphometrics. On top of that, *Chi/+* fish have highly mineralised vertebral body bone structures with no detectable osteoid (non-mineralised collagenous bone matrix), as confirmed by histology of non-demineralised specimens. Likewise, the osteoid thickness is reduced in the bone of human patients (31, 32) and murine OI models (33, 34). Moreover, hypermineralisation and bone brittleness are well documented in human patients (2, 4, 5, 29), OI mouse models (35) and OI zebrafish models (17, 36). Excess bone mineralisation related to increased dietary P intake or ageing is known to increase vertebral column malformations in WT zebrafish (22, 26). Hence, hypermineralisation alone can be considered a risk factor for bone deformities. In addition, OI bone is characterised by mutated collagen type I, which contributes to bone fragility. While mutations cannot be undone in OI patients or animal models, lowering the bone mineral content could possibly alleviate their skeletal defects. Recent findings on teleosts including WT zebrafish (22) and Atlantic salmon (23–25) have shown that low dietary P administration reduces the bone mineral content and promotes the formation of non-mineralised bone without causing vertebral column malformations. To assess the effects of reduced dietary P intake on the mutant bone phenotype, *Chi/+* were fed a low P diet (LP) from one month of age. *Chi/+* zebrafish under LP conditions (*Chi*/+_LP_) have reduced incidence of kyphosis and lordosis of the abdominal region of the vertebral column, and reduced shape variation of the caudal vertebral bodies. The reduced P intake restores the shape of vertebral bodies to a condition more similar to WT vertebral bodies. Moreover, the LP diet is shown to reduce the mineralisation of vertebral body endplates in some treated *Chi/+* fish and to restore the osteoid layer, that is absent in untreated mutants. It is known that the presence of an osteoid (non-mineralised collagen) has a beneficial effect on the mechanical properties of bone. The collagenous bone matrix itself is a very tough material that can withstand extreme mechanical forces and bend without fracturing. Examples for tough low mineralised bones that do not fracture are deer antlers and the bones of human infants (18), but also teleosts under reduced P intake show tough and deformable vertebral bodies with no signs of fractures (22, 24, 25). In contrast, high dietary P causes excess mineralisation and higher bone stiffness in teleosts (22, 24, 25), and results in reduced bone formation in humans (37, 38) and reduced ultimate strength (maximal load) in rats (39). Our findings suggest that the mineral content of *Chi/+* bone likely was reduced by the LP diet, which could explain the reduced incidence of vertebral column deformities and the restored shape of the vertebral bodies.

### *Chihuahua* Vertebrae Have Thin Bone Structures and Are Subjected to Compression Fractures

One of the most severe complications in OI patients are multiple compression fractures with collapse of the vertebral bodies (4, 29). Our findings show that *Chi/+* zebrafish suffer from the same type of complications. Several compression fractures were identified on whole mount specimens and on histological sections. Which are the factors predicted to contribute to compression fractures? The first possible factor for compression fractures is the poor quality of OI bone, caused by mutated collagen production and excess mineralisation. As discussed above, over-mineralised bone fractures easily; both patients and *Chi/+* zebrafish suffer from bone brittleness (5, 17). The second potential cause is reduced bone mass. Histological assessment of human biopsies showed that patients including children have thinner cortical bone and a reduced number of trabeculae in trabecular bone (4, 5). Likewise, two OI mice models (33, 34) and *Chi/+* zebrafish have thinner bone elements compared to WT, thin vertebral body endplates, thin bone elements in the vertebral body central region and thin bone trabeculae. Thus, hypermineralised vertebral bodies with poor trabecular bone are at mechanical disadvantage and are easily subjected to compression fractures (5). Bone with low mass and excess of minerals cannot easily withstand mechanical forces exerted along the axial skeleton. The third potential factor contributing to compression fractures is the weakness of the spinal ligaments and intervertebral discs. Early reports described that OI patients lack vertebral stability because of the laxity of the spinal ligaments (2, 4) and intervertebral discs (40, 41). Similarly, the *Col1a^Jrt^*/+ OI mouse model shows reduced cervical intervertebral space (42). Likewise, *Chi/+* zebrafish show evidence of weak intervertebral ligaments. Although on histological sections the ligaments of *Chi/+* fish display all the structural elements as in WT animals, *Chi/+* vertebral endplates are often shifted against each other along the dorsal-ventral axis. In humans weak ligaments fail to provide sufficient support to the vertebral column and allow the progressive degeneration of the deformity into a compression fracture (4). It can be assumed that less severe complications appear first and subsequently progress into a severe compression fracture. To support this hypothesis, patients show microfractures of the vertebral body growth plates (4, 43). During growth in humans, vertebral growth plate microfractures can progress into complicated lesions such as compression fractures (29, 44).

Human OI patients show evidence of fracture repair, but often fractures heal with deformities (45). We show similar findings in *Chi/+* zebrafish. Compression fractures in *Chi/+* are also subjected to fracture repair but the vertebral bodies remain deformed. Analysis of histological sections from different specimens suggests the identification of fractures in different stages of repair and allows for the tentative reconstruction of the steps involved in vertebral fracture healing. Like in mammals, *Chi/+* zebrafish fractures are initially stabilised by the formation of fibrocartilaginous calli, that subsequently become ossified. Despite the observation of fractures in different stages of repair, the design of our study does not allow to generate a timeline of fracture repair. In the goldfish (*Carassius auratus*), a cyprinid species like zebrafish, a bone fracture callus takes about 35 days to replace the fibrocartilaginous template (46). Thus, the presence of bone calli in compressed vertebral bodies of three months old *Chi/+* indicates that mutants likely developed vertebral compression fractures before the start of the experiment. This could explain why both *Chi/+* treated with the LP diet and untreated mutants show compression fractures. Compression fractures are therefore one of the most important complications during the initial stages of the disease, both in zebrafish and children affected by OI, and are crucial in the quick progression of the OI phenotype.

### *Chihuahua* Zebrafish Have Increased Bone Resorption and Reduced Bone Formation, but the LP Diet Improves the Bone Matrix

Bone from individuals with OI is characterised by an increased bone turnover rate and an increased number of osteoclasts, associated to an increased eroded surface compared to controls (47–49). Analogous findings derive from studies on two OI mouse models, the *Oim* (33) and *Brtl* (34) mice. Likewise, *Chi/+* zebrafish exhibit increased osteoclastic activity, as demonstrated by TRAP staining on histological sections. Resorption activity is increased at the endosteal surfaces of the neural and haemal arches compared to WT animals. TRAP activity extends also to other bone structures that are not subjected to resorption in WT zebrafish, such as the vertebral body endplates and the bone trabeculae that connect the vertebral body endplates (50). Trabecular bone surfaces display high osteoclast activity also in the *Oim* and *Brtl* mice (33, 34). The bone in zebrafish can be remodelled by multinucleated osteoclasts, but different from mammals, thin bony elements are typically resorbed by mononucleated cells (27). Flat, elongated mononucleated osteoclasts occur at endosteal surfaces, for example of neural and haemal arches (27, 50), as also observed in WT specimens in this study. Bone resorption in zebrafish is primarily related to the demands of allometric growth. The common type of mononucleated osteoclasts does not create deep resorption lacunae (27). TEM from *Chi/+* specimens showed osteoclasts with abundant electrondense cytoplasm characterised by several cytoplasmic vacuoles. These cells exhibit the typical ruffled border, indicative for active resorption of the bone matrix. Also osteoclasts in bone from the *Oim* mouse exhibited cellular changes. *Oim* cells have a larger diameter and exhibit three times the number of nuclei compared to osteoclasts in WT mice (51). The abundant cytoplasmic vacuoles of osteoclasts and the larger amount of TRAP-positive bone surfaces are indicative for increased resorptive activity in OI bone, both in *Chi/+* zebrafish and mouse models (34, 51). While endosteal bone resorption is increased in human OI patients, osteoblasts produce less new bone, which results in a reduced osteoid thickness, as discussed above. The decrease in bone formation in human patients, reviewed by Fedarko (49), is consistent with data from the *Oim* and *Brtl* mouse models (33, 34). *In vitro* studies have shown that osteoblasts from human patients (reviewed by 49) and murine models (52) show decreased synthesis, processing and matrix incorporation of collagen compared to controls. The production of mutated collagen type I in OI osteoblasts results in delayed collagen folding and excess post-translational modifications, that cause intracellular retention of defective collagen and endoplasmic reticulum (ER) stress (10). At an ultrastructural level, *Chi/+* zebrafish have osteoblasts in the vertebral growth zone with enlarged ER cisternae. Similar findings were previously described on osteoblasts from the caudal fin of adult *Chi/+* (16). Here we show that ER cisternae in *Chi/+* osteoblasts are filled with protein, likely mutated collagen type I. Intracellular collagen retention leads to collagen over modification and impairs collagen secretion in *Chi/+* (16) and in OI models (52). The extracellular collagen matrix is also impaired. In close proximity of the osteoblasts, the collagen fibrils in *Chi/+* fish are densely packed and lack the typical pattern of the osteoid collagen where the fibrils have a low density and are loosely arranged (53). In contrast, in *Chi/+* the fibrils in the bone matrix have a high degree of compactness that is typical of fully mineralised bone (53). These findings correlate well with the lack of osteoid seams in *Chi/+* and OI bone in general. It is widely accepted that insufficient osteoblast performance is at the basis of the severe OI phenotype. Thus, recent studies have targeted OI osteoblasts to relieve ER stress and improve cellular functions. The treatment with 4-phenylbutyrate, a chemical chaperone already approved by the FDA for urea cycle disorders, stimulates collagen secretion in murine OI osteoblasts *in vitro* (52), and improves the OI bone phenotype *in vivo* (16). In the present study, we show that two months of reduced dietary P administration partially rescue the OI phenotype in *Chi/+*. Mutants which received the LP diet show improved extracellular matrix with less dense collagen fibrils, indicative for a more typical osteoid. This finding together with the reduced incidence of vertebral column deformities and the rescue of the vertebral body shape, suggests that a reduced dietary P intake can alleviate the severe bone phenotype in juvenile *Chi/+* zebrafish.

## Materials and Methods

### Zebrafish Maintenance and Ethical Statement

Wild type AB (WT) and heterozygous *Chihuahua* (*col1a1a*^dc124/+^, *Chi/+*) zebrafish were bred in-house. The mutant *Chi/+* carries a heterozygous c.2207G>A mutation in *col1a1a* causing a p.G736D (G574D) substitution in the α1 chain of collagen type I (15). Zebrafish embryos were kept in petri dishes in fish water (1.2 mM NaHCO_3_, 0.01% instant ocean, 1.4 mM CaSO_4_, 0.0002% methylene blue) at 28°C until 7 days post-fertilisation (dpf), then housed in ZebTEC semi-closed recirculation housing systems (Techniplast, Buguggiate, Italy) at 28°C, pH 7.5 and conductivity 500 μS on a 14/10 light/dark cycle. Zebrafish from 7 to 21 dpf were fed three times a day alternating commercial dry food (ZM000, Zebrafish Management Ltd., Winchester, UK) and brine shrimp (Artemia cysts, Zebrafish Management Ltd., Winchester, UK). Fish were then fed for another week three times a day with a dry regular P diet (22), see also below), until 28 dpf, to adjust them to this type of dry feed. Starting from 28 dpf, WT were fed three times a day with a regular P diet for two months. At 28 dpf, *Chi/+* were randomly divided in two groups and fed three times a day with a ‘LP diet’ (low P content) or a regular P diet for two months (see below for details). Specimens were collected after two months of dietary treatment, euthanised by tricaine (3-amino benzoic acidethylester) overdose (0.3%) and fixed for further analyses as described below. The experiments were conducted in the centralised animal facility of the University of Pavia (Pavia, Italy). All animal studies were conducted in agreement with EU Directive 2010/63/EU for animals. The experimental protocol was approved by the Italian Ministry of Health (Approval animal protocol No. 260/2020-PR, 26 March 2020).

### Diet Composition

Diet composition and nutritional experiments are described in detail in (22). Briefly, the diets were formulated to have a total P content of 0.5% and 1.0%, termed low P (LP) diet and regular P diet, respectively (**Table 3**). Monoammonium phosphate (MAP) was used as dietary inorganic P supplement (54). In order to keep all diets equal in nutrients, except for P concentration, MAP replaced the inert filler diatomaceous earth (Diamol, Imerys, Denmark). The diets were formulated by SimplyFish AS (Stavanger, Norway, www.simplyfish.no) and produced by extrusion with subsequent crumbling to a suitable particle size by the Danish Technological Institute (Taastrup, Denmark, https://www.dti.dk). The P content of the product was verified at the University of Hohenheim (Stuttgart, Germany, https://www.uni-hohenheim.de) and determined with 5.04 g/kg diet and 9.84 g/kg diet for the low P and regular P diet, respectively (**Table 3**).

**Table 3:** Ingredients and chemical composition of the diets for zebrafish.

Ingredients (%)	Low P diet	Regular P diet
Rapeseed lecitin (Bergathin)	2.00	2.00
Krill meal	3.00	3.00
Wheat starch	18.77	18.77
Corn gluten meal	8.0	8.0
Wheat gluten meal	19.01	19.01
Soy protein concentrate	31.00	31.00
Capelin fish meal	5.00	5.00
Rapeseed oil	1.58	1.58
Peruvian fishoil	2.60	2.60
DL-Methionine	0.60	0.60
Biolys 54.6%	2.00	2.00
Lutavit C Aquastab 35%	0.10	0.10
Vitamin mix	0.50	0.50
Choline chloride 50%	1.50	1.50
Trace mineral mix (P free)	0.30	0.30
Monoammonium phosphate 26%	0.00	1.95
Diamol (diatomaceous earth)	4.00	2.05
Astaxanthin 10%	0.07	0.07
Total	100.00	100.00
**Chemical composition (g/kg)**		
Crude protein	497	508
Crude lipids	97	97
Crude ash	84	73
Calcium	4.84	4.79
Magnesium	1.96	1.98
Phosphorus	5.04	9.84

### X-Rays

X-rays of WT, *Chi/+* and *Chi/+* under LP diet were acquired with a Faxitron Mx-20 (Faxitron, Tucson, Arizona, USA) using 25 kV for 10 sec. The Kodak DirectView Elite CR System and k-Pacs software (Kodak, Rochester, New York, USA) were used for image digitalisation.

### Whole Mount Skeletal Staining

WT (n=26), *Chi/+* (n=20) and *Chi*/+_LP_ (n=18) were fixed for 24 h in 4% paraformaldehyde (PFA) in 1× phosphate-buffered saline (PBS) at C and were stained with Alizarin red S according to an established protocol (55). Fish were analysed and imaged using an Axio Zoom V16 stereomicroscope (Carl Zeiss, Oberkochen, Germany) with oblique illumination equipped with a 5MP CCD camera. Classification of the deformities was performed on whole mount-stained WT and mutants as defined by Martini et al. (56). Only the abdominal and caudal region of the vertebral column were analysed. The maximal curvature indices related to the abdominal and the caudal region of the vertebral column were calculated according to the method described by Marie-Hardy et al. (57). Briefly, the maximal kyphotic and lordotic indices were calculated in the sagittal plane of Alizarin red S stained specimens as the ratio (a/b) between the perpendicular distance from the axis in correspondence of the maximal curvature (segment ‘a’ in **Figure 1A**) and the standard length (described as the distance from the anterior most tip of the upper jaw to the posterior edge of the caudal peduncle where caudal fin rays insert (58), segment ‘b’ in **Figure 1A**). The same method but in the coronal plane was used to calculate the maximal scoliotic index. Moreover, for each group the following general metrics were calculated according to Martini et al. (56): the frequency (%) of specimens with at least one malformation, the total number of malformations identified, the average malformation load (total number of malformations diagnosed in a group/number of malformed individuals per group).

Lateral images of stained fish were used to quantitatively analyse mineralisation levels of vertebral endplates of WT, *Chi/+* and *Chi*/+_LP_ as described in (22). The first 5 caudal vertebral bodies (59) were considered for analysis in all specimens, following the established protocol (22). Briefly, the non-mineralised endplate was expressed as a percentage of the total non-mineralised endplate length over the total vertebral length. Vertebral endplates with a non-mineralised percentage value greater than 10% were classified as low mineralised, between 3% and 10% as intermediate mineralised, and less than 3% were considered high mineralised.

### Geometric Morphometrics of Vertebral Centra

Lateral images of whole mount Alizarin red S stained specimens were used to quantify the shape variation of the first 10 caudal vertebral centra (59) in WT (n=15), *Chi/+* (n=13) and *Chi*/+_LP_ (n=12) by means of landmark-based geometric morphometrics. The landmarks, defined as biologically homologous anatomical loci recognisable on all specimens in the study (60), were selected on the vertebral centra as represented in **Figure 2A**: landmarks number 1, 5, 6 and 10 on the vertebral endplates; landmarks number 2, 4, 7 and 9 on the anterior and posterior cone of the centrum; landmarks number 3 and 8 in the central region of the centrum. A similar configuration of landmarks has been applied in a vertebral fracture assessment study in human patients (61). 2D landmarks were extracted from digital images using Fiji (NIH, Bethesda, Maryland, USA) and digitised in the same order for all vertebral centra analysed. Procrustes superimposition of digitised landmarks and visualisation of shape variations were performed using Past4.04 software (62). Principal component analysis was performed using PCAGen8 software as described in (60).

### Histology and Bone Histomorphometry

WT (n=4), *Chi/+* (n=4) and *Chi*/+_LP_ (n=5) were fixed for 24 h in 2.5% PFA, 1.5% glutaraldehyde, 0.1 M sodium cacodylate buffer (pH 7.4) and 0.001% CaCl_2_ at 4°C, decalcified in 0.1 M EDTA for 14 days at 4°C and embedded in glycol methacrylate (27). Sagittal 2 μm sections were cut on a Microm HM360 (Marshall Scientific, Hampton, New Hampshire, USA) automated microtome and were stained with toluidine blue (0.5% toluidine blue, 1% Na_2_B_4_O_7_ in demineralised H_2_O (dH_2_O), pH 9 for 15 sec), rinsed in dH_2_O and mounted with DPX. Images were acquired using an Axio Imager-Z1 microscope (Carl Zeiss, Oberkochen, Germany) equipped with an Axiocam 503 colour camera (Carl Zeiss, Oberkochen, Germany). Bone structure histomorphometry was analysed on images of toluidine blue stained sections of the middle plane of the vertebral column. Thickness of bone structures in the endplates, in the central region and in the trabeculae of 5 to 10 vertebral centra per specimen were measured using Fiji (NIH, Bethesda, Maryland, USA) (see **Figure 3A** for location). The mean values were considered for analysis.

For mineral detection on histological sections, WT and mutant zebrafish were selected based on X-rays to be representative for the phenotype. WT (n=1), *Chi/+* (n=1) and *Chi*/+_LP_ (n=1) were fixed as described above and embedded in glycol methacrylate without carrying out decalcification. Sections of 2 μm were stained according to the Von Kossa/Van Gieson protocol (63). Images were acquired using an Axio Imager-Z1 microscope (Carl Zeiss, Oberkochen, Germany) equipped with a 5MP CCD camera.

### Transmission Electron Microscopy

WT (n=1), *Chi/+* (n=1) and *Chi*/+_LP_ (n=1) were selected based on X-rays to be representative for the phenotype, fixed and decalcified as described above for histology, and embedded in epon epoxy medium (64). Semi-thin 1 μm sagittal sections were cut on a Microm HM360 microtome (Marshall Scientific, Hampton, New Hampshire, USA), stained with toluidine blue at pH 9 for 2 min, rinsed with dH_2_O and mounted with DPX. For TEM analysis, ultrathin sections (about 70 nm) of the middle plane of the vertebral column were prepared on an UltracutE ultramicrotome (Reichert-Jung, Buffalo, New York, USA), contrasted with uranyl acetate and lead citrate and analysed with a Jeol JEM 1010 transmission electron microscope (Jeol Ltd., Tokyo, Japan) operating at 60 kV. Microphotographs were taken with a Veleta camera (Emsis, Muenster, Germany).

### Enzyme Histochemistry

WT (n=2), *Chi/+* (n=2) and *Chi*/+_LP_ (n=2) were fixed in 4% PFA in 1× PBS, pH 7.4, for 1 h at RT and decalcified in 4% PFA, 10% EDTA, pH 7.4 for 14 days at 4°C. Specimens were embedded in glycol methacrylate (27). Sagittal 5 μm sections were cut on a Microm HM360 (Marshall Scientific, Hampton, New Hampshire, USA) automated microtome and demonstration of tartrate-resistant acid phosphatase (TRAP) was adapted from (27). Briefly, sections were pre-incubated at 37°C for 45 min in 50 mL acetate buffer (0.1 M sodium acetate, 50 mM L(+) di-sodium tartrate dehydrate, pH adjusted to 5.5 with acetic acid) to which is added 0.5 mL of enzyme substrate solution (2% Naphtol AS TR phosphate dissolved in ethylene glycol mono-butyl ether). Shortly before use, 1 mL of pararosaniline solution (4% pararosaniline chloride (CI. 42500) in 7% HCl solution) was mixed to 1 mL of fresh 5% sodium nitrite and, after hexazotiation, was added to the acetate-enzyme substrate solution. Enzymatic reaction took place in 30-60 min at 37°C. Subsequently, slides were rinsed in dH_2_O, counterstained with Meyers haematoxylin for 10 min, rinsed in running tap water for 10 min, flushed in dH_2_O, dried at 40°C and mounted with DPX.

### Statistical Analysis

Quantitative variables are expressed as mean ± standard deviation, categories are expressed as percentages. Statistical analysis was performed using Past4.04 software (62). Differences in the occurrence of vertebral column deformities and in bone mineralisation levels were evaluated by means of Chi-square test followed by Bonferroni correction. Comparison of the maximal curvature indices was based on the non-parametric Mann-Whitney test. Differences in the thickness of bone structures were evaluated by means of Mann-Whitney non-parametric test followed by Bonferroni correction. For principal component analysis of geometric morphometrics data, significant differences in principal component 1 and principal component 2 were obtained using PCAGen8 software as described in (60) using Chi-square test (paired tests, WT-*Chi/+* and WT-*Chi*/+_LP_). A p value less than 0.05 was considered significant.

## Data Availability Statement

The raw data supporting the conclusions of this article will be made available by the authors, without undue reservation.

## Ethics Statement

The animal study was reviewed and approved by Italian Ministry of Health.

## Author Contributions

SC, PEW, and AF designed the study. SC carried out the research. DL carried out TEM. WK designed the diets. SC, PW, and AH analysed the results and drafted the manuscript. PW and AH obtained the funding. All authors contributed to the article and approved the submitted version.

## Funding

SC and PEW acknowledge funding by the European Union’s Horizon 2020 Research and Innovation Programme under the Marie Skłodowska-Curie grant agreement No 766347 (Biomedaqu) and by Ghent University, Bijzonder Onderzoeksfonds grant code BOF.ITN.2021.0012.01. AH acknowledges Bijzonder Onderzoeksfonds grant from Ghent University in the frame of Concerted Research Actions funding No BOFGOA2021000407.

## Conflict of Interest

WK was employed by the company SimplyFish AS.

The remaining authors declare that the research was conducted in the absence of any commercial or financial relationships that could be construed as a potential conflict of interest.

## Publisher’s Note

All claims expressed in this article are solely those of the authors and do not necessarily represent those of their affiliated organizations, or those of the publisher, the editors and the reviewers. Any product that may be evaluated in this article, or claim that may be made by its manufacturer, is not guaranteed or endorsed by the publisher.

## References

[1] Forlino A Marini JC. Osteogenesis Imperfecta. Lancet (2016) 387:1657–71. doi: 10.1016/S0140-6736(15)00728-X PMC7384887 26542481

[2] Sillence DO Senn A Danks DM. Genetic Heterogeneity in Osteogenesis Imperfecta. J Med Genet (1979) 16:101–16. doi: 10.1136/jmg.16.2.101 PMC1012733 458828

[3] Marini JC Forlino A Bächinger HP Bishop NJ Byers PH Paepe A. Osteogenesis Imperfecta. Nat Rev Dis Primers (2017) 3:17052. doi: 10.1038/nrdp.2017.52 28820180

[4] Benson DR Newman DC. The Spine and Surgical Treatment in Osteogenesis Imperfecta. Clin Orthop Relat Res (1981) 159:147–53. doi: 10.1097/00003086-198109000-00020 7285452

[5] Zeitlin L Fassier F Glorieux FH. Modern Approach to Children With Osteogenesis Imperfecta. J Pediatr Orthop B (2003) 12:77–87. doi: 10.1097/01.bpb.0000049567.52224.fa 12584489

[6] Ben Amor IM Roughley P Glorieux FH Rauch F. Skeletal Clinical Characteristics of Osteogenesis Imperfecta Caused by Haploinsufficiency Mutations in COL1A1. J Bone Miner Res (2013) 28:2001–7. doi: 10.1002/jbmr.1942 23529829

[7] Wallace MJ Kruse RW Shah SA. The Spine in Patients With Osteogenesis Imperfecta. J Am Acad Orthop Surg (2017) 25:100–9. doi: 10.5435/JAAOS-D-15-00169 28009707

[8] Castelein RM Hasler C Helenius I Ovadia D Yazici M EPOS Spine Study Group. Complex Spine Deformities in Young Patients With Severe Osteogenesis Imperfecta: Current Concepts Review. J Child Orthop (2019) 13:22–32. doi: 10.1302/1863-2548.13.180185 30838072 PMC6376432

[9] Marini JC Forlino A Cabral WA Barnes AM San Antonio JD Milgrom S. Consortium for Osteogenesis Imperfecta Mutations in the Helical Domain of Type I Collagen: Regions Rich in Lethal Mutations Align With Collagen Binding Sites for Integrins and Proteoglycans. Hum Mutat (2007) 28:209–21. doi: 10.1002/humu.20429 PMC4144349 17078022

[10] Ishikawa Y Bächinger HP. A Molecular Ensemble in the rER for Procollagen Maturation. Biochim Biophys Acta (2013) 1833:2479–91. doi: 10.1016/j.bbamcr.2013.04.008 23602968

[11] Huysseune A. “Skeletal System”. In: Ostrander GK, editor. The Laboratory Fish. Part 4 Microscopic Functional Anatomy. San Diego: Academic Press (2000). p. 307–17.

[12] Witten PE Harris MP Huysseune A Winkler C. Small Teleost Fish Provide New Insights Into Human Skeletal Diseases. Methods Cell Biol (2017) 138:321–46. doi: 10.1016/bs.mcb.2016.09.001 28129851

[13] Tonelli F Bek JW Besio R De Clercq A Leoni L Salmon P. Zebrafish: A Resourceful Vertebrate Model to Investigate Skeletal Disorders. Front Endocrinol (2020) 11:489. doi: 10.3389/fendo.2020.00489 PMC7416647 32849280

[14] Dietrich K Fiedler IAK Kurzyukova A López-Delgado AC McGowan LM Geurtzen K. Skeletal Biology and Disease Modeling in Zebrafish. J Bone Miner Res (2021) 36:436–58. doi: 10.1002/jbmr.4256 33484578

[15] Fisher S Jagadeeswaran P Halpern ME. Radiographic Analysis of Zebrafish Skeletal Defects. Dev Biol (2003) 264:64–76. doi: 10.1016/s0012-1606(03)00399-3 14623232

[16] Gioia R Tonelli F Ceppi I Biggiogera M Leikin S Fisher S. The Chaperone Activity of 4PBA Ameliorates the Skeletal Phenotype of Chihuahua, a Zebrafish Model for Dominant Osteogenesis Imperfecta. Hum Mol Genet (2017) 26:2897–911. doi: 10.1093/hmg/ddx171 PMC5886106 28475764

[17] Fiedler IAK Schmidt FN Wölfel EM Plumeyer C Milovanovic P Gioia R. Severely Impaired Bone Material Quality in Chihuahua Zebrafish Resembles Classical Dominant Human Osteogenesis Imperfecta. J Bone Miner Res (2018) 33:1489–99. doi: 10.1002/jbmr.3445 29665086

[18] Currey JD. The Many Adaptations of Bone. J Biomech (2003) 36:1487–95. doi: 10.1016/S0021-9290(03)00124-6 14499297

[19] Veis A. “Mineralization in Organic Matrix Frameworks”. In: Dove PM Yoreo JJ Weiner SW, editors. Biomineralization. Reviews in Mineralogy and Geochemistry. Chantilly, VA: Mineralogical Society of America (2003). p. 249–89.

[20] Wilton TJ Hosking DJ Pawley E Stevens A Harvey L. Osteomalacia and Femoral Neck Fractures in the Elderly Patient. J Bone Joint Surg Br (1987) 69:388–90. doi: 10.1302/0301-620X.69B3.3584190 3584190

[21] Guglielmi G Muscarella S Bazzocchi A. Integrated Imaging Approach to Osteoporosis: State-of-the-Art Review and Update. Radiographics (2011) 31:1343–64. doi: 10.1148/rg.315105712 21918048

[22] Cotti S Huysseune A Koppe W Rücklin M Marone F Wölfel EM. More Bone With Less Minerals? The Effects of Dietary Phosphorus on the Post-Cranial Skeleton in Zebrafish. Int J Mol Sci (2020) 21:5429. doi: 10.3390/ijms21155429 PMC7432380 32751494

[23] Witten PE Owen MA Fontanillas R Soenens M McGurk C Obach A. A Primary Phosphorus-Deficient Skeletal Phenotype in Juvenile Atlantic Salmon Salmo Salar: The Uncoupling of Bone Formation and Mineralization. J Fish Biol (2016) 88:690–708. doi: 10.1111/jfb.12870 26707938 PMC4784172

[24] Witten PE Fjelldal PG Huysseune A McGurk C Obach A Owen MAG. Bone Without Minerals and its Secondary Mineralization in Atlantic Salmon. J Exp Biol (2019) 222:jeb188763. doi: 10.1242/jeb.188763 30573664

[25] Drábiková L Fjelldal PG De Clercq A Yousaf MN Morken T McGurk C. Vertebral Column Adaptations in Juvenile Atlantic Salmon Salmo Salar, L. As a Response to Dietary Phosphorus. Aquaculture (2021) 541:736776. doi: 10.1016/j.aquaculture.2021.736776

[26] Kague E Turci F Newman E Yang Y Brown KR Aglan MS. 3D Assessment of Intervertebral Disc Degeneration in Zebrafish Identifies Changes in Bone Density That Prime Disc Disease. Bone Res (2021) 9:39. doi: 10.1038/s41413-021-00156-y 34465741 PMC8408153

[27] Witten PE Hansen A Hall BK. Features of Mono- and Multinucleated Bone Resorbing Cells of the Zebrafish *Danio Rerio* and Their Contribution to Skeletal Development, Remodeling, and Growth. J Morphol (2001) 250:197–207. doi: 10.1002/jmor.1065 11746460

[28] Witten PE Hall BK. “The Ancient, Segmented, Active and Permanent Notochord”. In: Pradel A Denton JSS Janvier P, editors. Ancient Fishes and Their Living Relatives: A Tribute to John G. Maisey. München, Germany: Verlag Dr. Friedrich Pfeil (2021). p. 215–24.

[29] Shah SA Wallace MJ. “Osteogenesis Imperfecta in the Spine”. In: Kruse RW, editor. Osteogenesis Imperfecta. A Case-Based Guide to Surgical Decision-Making and Care. Cham, Switzerland: Springer (2020). p. 221–30. doi: 10.1007/978-3-030-42527-2

[30] Benson DR Donaldson DH Millar EA. The Spine in Osteogenesis Imperfecta. J Bone Joint Surg Am (1978) 60:925–9. doi: 10.2106/00004623-197860070-00009 701340

[31] Stöß H Pontz BF Pesch H-J Ott R. Heterogeneity of Osteogenesis Imperfecta. Biochemical and Morphological Findings in a Case of Type III According to Sillence. Eur J Pediatr (1986) 145:34–9. doi: 10.1007/BF00441849 3732329

[32] Iwamoto J Takeda T Ichimura S. Increased Bone Resorption With Decreased Activity and Increased Recruitment of Osteoblasts in Osteogenesis Imperfecta Type I. J Bone Miner Metab (2002) 20:174–9. doi: 10.1007/s007740200025 11984701

[33] Kalajzic I Terzic J Rumboldt Z Mack K Naprta A Ledgard F. Osteoblastic Response to the Defective Matrix in the Osteogenesis Imperfecta Murine (Oim) Mouse. Endocrinology (2002) 143:1594–601. doi: 10.1210/endo.143.5.8807 11956140

[34] Uveges TE Collin-Osdoby P Cabral WA Ledgard F Goldberg L Bergwitz C. Cellular Mechanism of Decreased Bone in Brtl Mouse Model of OI: Imbalance of Decreased Osteoblast Function and Increased Osteoclasts and Their Precursors. J Bone Miner Res (2008) 23:1983–94. doi: 10.1359/jbmr.080804 PMC2686922 18684089

[35] Enderli TA Burtch SR Templet JN Carriero A. Animal Models of Osteogenesis Imperfecta: Applications in Clinical Research. Orthop Res Rev (2016) 8:41–55. doi: 10.2147/ORR.S85198 30774469 PMC6209373

[36] Tonelli F Cotti S Leoni L Besio R Gioia R Marchese L. Crtap and *P3h1* Knock Out Zebrafish Support Defective Collagen Chaperoning as the Cause of Their Osteogenesis Imperfecta Phenotype. Matrix Biol (2020) 90:40–60. doi: 10.1016/j.matbio.2020.03.004 32173581

[37] Kemi VE Kärkkäinen MUM Lamberg-Allardt CJE. High Phosphorus Intakes Acutely and Negatively Affect Ca and Bone Metabolism in a Dose-Dependent Manner in Healthy Young Females. Br J Nutr (2006) 96:545–52. doi: 10.1079/BJN20061838 16925861

[38] Dermience M Lognay G Mathieu F Goyens P. Effects of Thirty Elements on Bone Metabolism. J Trace Elem Med Biol (2015) 32:86–106. doi: 10.1016/j.jtemb.2015.06.005 26302917

[39] Huttunen MM Tillman I Viljakainen HT Tuukkanen J Peng Z Pekkinen M. High Dietary Phosphate Intake Reduces Bone Strength in the Growing Rat Skeleton. J Bone Miner Res (2007) 22:83–92. doi: 10.1359/jbmr.061009 17042736

[40] Brailsford JF. Osteogenesis Imperfecta. Br J Radiol (1943) 16:129–36. doi: 10.1259/0007-1285-16-185-129

[41] Watanabe G Kawaguchi S Matsuyama T Yamashita T. Correlation of Scoliotic Curvature With Z-Score Bone Mineral Density and Body Mass Index in Patients With Osteogenesis Imperfecta. Spine (2007) 32:E488–94. doi: 10.1097/BRS.0b013e31811ec2d9 17762282

[42] Abdelaziz DM Abdullah S Magnussen C Ribeiro-da-Silva A Komarova SV Rauch F. Behavioral Signs of Pain and Functional Impairment in a Mouse Model of Osteogenesis Imperfecta. Bone (2015) 81:400–6. doi: 10.1016/j.bone.2015.08.001 26277094

[43] McKusick VA. Heritable Disorders of Connective Tissue. 4th ed. Mosby CV, editor. St Louis: Mosby Company (1972).

[44] Ishikawa S Kumar SJ Takahashi HE Homma M. Vertebral Body Shape as a Predictor of Spinal Deformity in Osteogenesis Imperfecta. J Bone Joint Surg Am (1996) 78:212–9. doi: 10.2106/00004623-199602000-00007 8609111

[45] McGreal C Bober MB. “Patient Evaluation and Medical Treatment for Osteogenesis Imperfecta”. In: Kruse RW, editor. Osteogenesis Imperfecta. A Case-Based Guide to Surgical Decision-Making and Care. Cham, Switzerland: Springer (2020). p. 11–9. doi: 10.1007/978-3-030-42527-2

[46] Moss ML. Studies of the Acellular Bone of Teleost Fish. II. Response to Fracture Under Normal and Acalcemic Conditions. Acta Anat (1962) 48:46–60 2975–3015. doi: 10.1002/dvdy.22113 14476543

[47] Baron R Gertner JM Lang R Vignery A. Increased Bone Turnover With Decreased Bone Formation by Osteoblasts in Children With Osteogenesis Imperfecta Tarda. Pediatr Res (1983) 17:204–7. doi: 10.1203/00006450-198303000-00007 6835724

[48] Rauch F Travers R Parfitt AM Glorieux FH. Static and Dynamic Bone Histomorphometry in Children With Osteogenesis Imperfecta. Bone (2000) 26:581–9. doi: 10.1016/s8756-3282(00)00269-6 10831929

[49] Fedarko NS. “Osteoblast/osteoclast Development and Function in Osteogenesis Imperfecta”. In: Shapiro JR Byers PH Glorieux FH Sponseller PD, editors. Osteogenesis Imperfecta. A Translational Approach to Brittle Bone Disease. San Diego: Academic Press (2014). p. 45–56. doi: 10.1016/B978-0-12-397165-4.00005-8

[50] Witten PE Huysseune A. A Comparative View on Mechanisms and Functions of Skeletal Remodelling in Teleost Fish, With Special Emphasis on Osteoclasts and Their Function. Biol Rev Camb Philos Soc (2009) 84:315–46. doi: 10.1111/j.1469-185X.2009.00077.x 19382934

[51] Zhang H Doty SB Hughes C Dempster D Camacho NP. Increased Resorptive Activity and Accompanying Morphological Alterations in Osteoclasts Derived From the Oim/Oim Mouse Model of Osteogenesis Imperfecta. J Cell Biochem (2007) 102:1011–20. doi: 10.1002/jcb.21337 PMC2944034 17668424

[52] Garibaldi N Contento BM Babini G Morini J Siciliani S Biggiogera M. Targeting Cellular Stress In Vitro Improves Osteoblast Homeostasis, Matrix Collagen Content and Mineralization in Two Murine Models of Osteogenesis Imperfecta. Matrix Biol (2021) 98:1–20. doi: 10.1016/j.matbio.2021.03.001 33798677 PMC11162743

[53] Bonucci E. Biological Calcification. Normal and Pathologicalpprocesses in the Early Stages. Berlin, Germany: Springer-Verlag (2007).

[54] Morales GA Azcuy RL Casaretto ME Márquez L Hernández AJ Gómez F. Effect of Different Inorganic Phosphorus Sources on Growth Performance, Digestibility, Retention Efficiency and Discharge of Nutrients in Rainbow Trout (*Oncorhynchus Mykiss*). Aquaculture (2018) 495:568–74. doi: 10.1016/j.aquaculture.2018.06.036

[55] Taylor WR Van Dyke GC. Revised Procedures for Staining and Clearing Small Fishes and Other Vertebrates for Bone and Cartilage Study. Cybium (1985) 9:107–19.

[56] Martini A Huysseune A Witten PE Boglione C. Plasticity of the Skeleton and Skeletal Deformities in Zebrafish (*Danio Rerio*) Linked to Rearing Density. J Fish Biol (2020) 98:971–86. doi: 10.1111/jfb.14272 32010967

[57] Marie-Hardy L Khalifé M Slimani L Pascal-Moussellard H. Computed Tomography Method for Characterising the Zebrafish Spine. Orthop Traumatol Surg Res (2019) 105:361–7. doi: 10.1016/j.otsr.2018.12.008 30799173

[58] Parichy DM Elizondo MR Mills MG Gordon TN Engeszer RE. Normal Table of Postembryonic Zebrafish Development: Staging by Externally Visible Anatomy of the Living Fish. Dev Dyn (2009) 238:2975–3015. doi: 10.1002/dvdy.22113 19891001 PMC3030279

[59] Bird NC Mabee PM. Developmental Morphology of the Axial Skeleton of the Zebrafish, *Danio Rerio* (Ostariophysi: Cyprinidae). Dev Dyn (2003) 228:337–57. doi: 10.1002/dvdy.10387 14579374

[60] Webster M Sheets H. “A Practical Introduction to Landmark-Based Geometric Morphometrics”. In: Alroy J Hunt G, editors. The Paleontological Society Papers. Cambridge: Cambridge University Press (2010). p. 163–88. doi: 10.1017/S1089332600001868

[61] Jager PL Slart RH Webber CL Adachi JD Papaioannou AL Gulenchyn KY. Combined Vertebral Fracture Assessment and Bone Mineral Density Measurement: A Patient-Friendly New Tool With an Important Impact on the Canadian Risk Fracture Classification. Can Assoc Radiol J (2010) 61:194–200. doi: 10.1016/j.carj.2009.12.012 20199851 PMC5102695

[62] Hammer Ø Harper DAT Ryan PD. Past: Paleontological Statistics Software Package for Education and Data Analysis. Palaeontol Electron (2001) 4:1–9.

[63] Humason GL Presnell JK Schreibman MP. Humason’s Animal Tissue Techniques. Baltimore, MD, USA: Johns Hopkins University Press (1997).

[64] Spurr AR. A Low-Viscosity Epoxy Resin Embedding Medium for Electron Microscopy. J Ultrastruct Res (1969) 26:31–43. doi: 10.1016/S0022-5320(69)90033-1 4887011

